# Eosinophilic Pleural Effusion: A Rare Complication of Extracorporeal Shock Wave Lithotripsy

**DOI:** 10.1155/2013/147986

**Published:** 2013-07-07

**Authors:** Maral Mokhtari, Perikala Vijayananda Kumar, Mohammad-Ali Ghayumi

**Affiliations:** ^1^Pathology Department, Shiraz University of Medical Sciences, Shiraz 71345-1864, Iran; ^2^Internal Medicine Department, Shiraz University of Medical Sciences, Shiraz 71345-1864, Iran

## Abstract

*Background*. Extracorporeal shock wave lithotripsy has been widely used to treat renal stones. The procedure is relatively safe with minor complications. *Case*. The patient is a 32-year-old man who presented with left sided pleural effusion after extracorporeal shock wave lithotripsy. *Results*. The pleural effusion study revealed an exudative fluid rich in eosinophils (30%). So, the diagnosis of eosinophilic pleural effusion as a complication of lithotripsy was made. *Conclusion*. Extracorporeal shock wave lithotripsy should be regarded as an etiology of unexplained eosinophilic pleural effusion after this procedure.

## 1. Introduction

Extracorporeal shock wave lithotripsy (ESWL) has been widely used to treat renal stones since 1980 [1, 2]. This procedure is noninvasive and relatively safe but short, and long-term complications are on record in about 3–7% of patients [2]. Pulmonary complications including contusion, cavitation, pulmonary edema, and pleural effusion have been reported mainly due to malposition of ESWL device [3–5]. Eosinophilic pleural effusion (EPE) is defined as an effusion containing at least 10% eosinophils. It occurs in different conditions [6–8]. Herein, we report a case of EPE following ESWL.

## 2. Case

**Figure 1:**
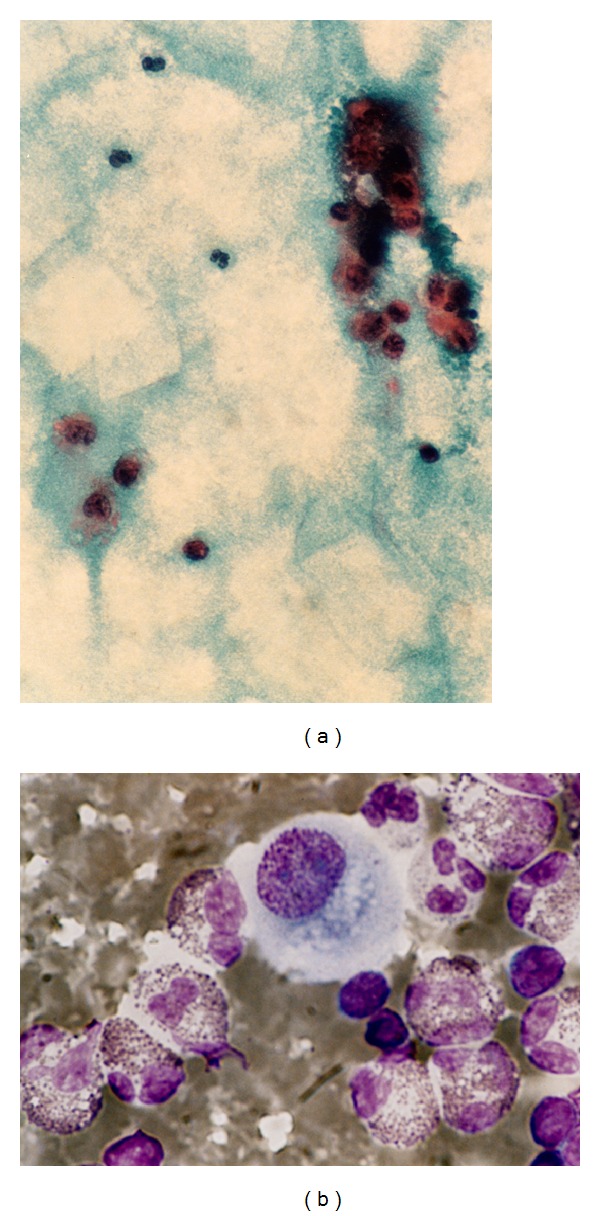
(a) and (b): pleural effusion differential count shows more than 10% eosinophils, Papanicolaou and Wright Giemsa stain, ×400, and oil immersion.

The patient is a 32-year-old man who was referred to Faghihi hospital affiliated to Shiraz University of Medical Sciences with the complaint of cough and dyspnea, which had started acutely. He denied fever, chills, and weight loss. His past medical history was unremarkable except for treating renal stone (left sided) by ESWL five days prior to admission. His physical examination showed stable vital signs. Decreased breathing sound and dullness on percussion were found over the left lung base. A chest X-ray was ordered which revealed left sided pleural effusion. Thoracentesis was performed. Aspirated fluid was yellow and slightly cloudy. The biochemical tests on pleural effusion showed glucose; 85 mg/dL, protein; 6.5 g/dL, lactate dehydrogenase; 520 IU/L (normal 200–400 IU/L), PH; 7.3, and creatinine; 1.0 mg/dL. The same parameters were measured in the patient's serum (glucose; 90 mg/dL, protein; 7 g/dL, LDH; 210 IU/L, PH; 7.4, and creatinine; 1.2). According to Light's criteria, the effusion was classified as exudative. Urinothorax was excluded based on creatinine and PH values. The fluid differential count showed 1000/mm^3^ red blood cells and 32500/mm^3^ leukocytes (30% eosinophils, 10% neutrophils, 40% lymphocytes, and 20% monocytes) (Figures 1(a) and 1(b)). Gram and acid-fast stains were negative, and no malignant cells were identified in the cytologic study. Complete blood count with differentials showed neutrophils 55%, lymphocytes 35%, monocytes 6%, and eosinophils 4%.  Table 1 summarizes the laboratory data of the patient. The diagnosis of EPE was made. No evidence of infections, drug reactions, malignancies, autoimmune disorders, and allergic diseases was found as a specific cause of PEP despite the extensive search so the effusion was considered as being related to ESWL. So, steroid was started. Hospital course was uneventful, the patient was discharged after three days, and the effusion was resorbed eventually during the next month and did not recur. 

## 3. Discussion

ESWL procedure may cause some pulmonary complications including contusion, cavitation, pulmonary edema, and pleural effusion mainly due to malposition of ESWL device. We report EPE following ESWL. EPE is defined as an effusion containing at least 10% eosinophils. Its incidence ranges between 5 and 16% of pleural effusions. This can be associated with various conditions including malignancies (solid organ and hematogenous), infections, and posttraumatics after medical or surgical intervention in the settings of spontaneous pneumothorax and hemothorax, autoimmune disorders, drug reactions, miscellaneous disorders, and idiopathic forms [6–8]. The pathogenesis of EPE may be due to cytokine release such as IL-5, granulocyte/macrophage colony stimulating factor, and IL-3, which increase production and survival of eosinophils [9, 10]. Malposition of ESWL device may cause pleural irritation followed by release of previously mentioned cytokine, leading to pleural effusion. 

Urinothorax may also occur following ESWL. This condition is defined as accumulation of urine in the pleural space. It usually occurs secondary to obstructive uropathy and rarely after retroperitoneal inflammatory or malignant disease, renal biopsy, trauma, percutaneous, and endoscopic renal or ureteral intervention [3, 4]. It is usually transudative and smells like urine. Biochemical tests may be helpful to differentiate this problem with other causes of pleural effusion which show low PH (depending on urine PH) with higher serum creatinine level. Because EPE occurs in the settings of various conditions, the next task after diagnosis of EPE is to search for finding the etiology of EPE because the patients' prognosis and their therapy are directly related to the cause of EPE, and ESWL should be considered as an etiology of unexplained EPE following this procedure.

## Figures and Tables

**Table 1:** Pleural fluid and serum biochemical data and differential count.

Test	Pleural fluid	Serum
Total protein	6.5 g/L	7 g/L
Glucose	85 mg/dL	90 mg/dL
LDH*	520 IU/L	210 IU/L
Creatinine	1 mg/dL	1.2 mg/dL
PH	7.3	7.4
Differential count		
Eosinophils	30%	4%
Neutrophils	10%	55%
Lymphocytes	40%	35%
Monocytes	20%	6%

*Lactate dehydrogenase.
